# Environmental, Ecological, and Economic Benefits of Biofuel Production Using a Constructed Wetland: A Case Study in China

**DOI:** 10.3390/ijerph16050827

**Published:** 2019-03-07

**Authors:** Dong Liu, Changxin Zou, Mengjia Xu

**Affiliations:** Ministry of Ecology and Environment, Nanjing Institute of Environmental Sciences, Nanjing 210042, China; liudong@nies.org (D.L.); zcx@nies.org (C.Z.)

**Keywords:** biomass energy, life cycle, cellulosic ethanol

## Abstract

Here we show a constructed wetland (CW), a viable alternative wastewater treatment system, be used to produce biofuels from biomass by using nitrogen contained in domestic wastewater. We summarize the potential biomass yield evaluated as cellulosic ethanol bioenergy production, and combine the life cycle analysis with a mass balance approach to estimate the energetic, environmental, and economic performance of a CW biofuel system. The results showed that the annual aboveground biomass yield of a CW in Zhoushan, Zhejiang Province, China, averaged 37,813 kg ha^−1^ year^−1^ as the by-product of treating waste N, which is about one order of magnitude larger than traditional biofuel production systems. The biomass yield in the Zhoushan CW system had life cycle environment benefits of 8.8 Mg (1 Mg = 10^6^ g) CO_2_ equivalent ha^−1^ year^−1^ of greenhouse gas emission reduction. The CW in Zhoushan had a net energy gain of 249.9 GJ (1 GJ = 10^9^ J) ha^−1^ year^−1^ while the wastewater treatment plant (WTP) consumes 7442.5 GJ ha^−1^ year^−1^. Moreover, the CW reduced greenhouse gas emissions to 2714 times less than that of the WTP. The CW also provided various ecosystem services, such as regional climate regulation and habitat conservation. We suggest that the potential use of a CW as biofuel production and carbon sequestration via nitrogen-negative input can be explored more widely in the future.

## 1. Introduction

Each year, the global population produces ~20 Tg of nitrogen (N) in human waste [1], which undergoes treatment and requires energy consumption. The wastewater treatment plant (WTP), which is currently the mainstream method for wastewater treatment, treats N waste and consumes a large amount of energy, thus making the overall life cycle accounting a concern for the production and removal of nitrogen, which could double energy consumption. Although N production and consumption causes pollution and an energy crisis, people seek biofuels for reducing pollution and energy usage. Unfortunately, current biofuel productions are also N-fertilizer dependent, such as the Brazilian program of sugarcane ethanol [2], the US program of corn grain ethanol, and soybean biodiesel [3]. It suggests that biofuel is not as clean as it is expected to be. People then seek second-generation biofuels, which provide greater benefits if their biomass feedstocks were producible with low agricultural input (i.e., less fertilizer, pesticide, and energy), such as cellulosic ethanol from switchgrass (*Panicum virgatum* L.) [4] and carbon-negative biofuels from low-input high-diversity (LIHD) grassland biomass [5,6]. Nonetheless, second-generation biofuels would still be N-dependent. This furthers the idea that using municipal and industrial wastes as biofuel feedstock, which are frequently rich in nutrients, means that there is no extra need for N supply in biofuel production [7].

Here, we propose using waste N via constructed wetlands (CW) for biofuel production. In a CW, the processes that affect the removal and retention of N during wastewater treatment includes microbial nitrification/denitrification, plant uptake, and substrate absorption [8]. Biomass is produced together with the pollutant removal from where wastewater occurs [9,10], and thus can be harvested and used as biofuel production. Efforts to develop CW systems for wastewater treatment have been undertaken since the 1970s in Europe and North America, and have been increasingly established in developing countries in recent years [11,12]. Most studies were aimed primarily at the pollutant removal efficiency [13], the biomass primary productivity of CW plants [14], the microbial community in substrates [15], the effects of plants on N removal [16], and the ecosystem service value assessment for a CW [17]. However, the concept of biomass production used as a biofuel is rarely reported [10,18]. The potential of CW technology can simultaneously solve problems of eutrophication and the energy crisis. Nevertheless, there still exists no feasible cases to support such an idea and no quantity analysis for its biofuel production. 

In this study, we use energy production to evaluate the energy efficiency of cellulosic ethanol derived from the CW biomass. Here, we take into account the expansive system boundaries for biomass energy inputs from plant seedling growth to harvest and convert them to biofuels. The output of biofuel production examines the conversion of biomass to cellulosic ethanol biofuel. Finally, for the purpose of examining this issue in a contrasting context with the ‘have to’ cost of wastewater treatment, we make simplifying assumptions that take the WTP as the baseline in estimating the effects of net energy gain, greenhouse gas (GHG) emissions, and the social-economic benefits for CW.

## 2. Materials and Methods 

### 2.1. Wetland Location and Design

A 1000 m^2^ CW system was built in 2005 at Zhujiajian (29°53′ N, 122°23′ E), Zhoushan City, Zhejiang Province in Southeast China. The goal was to remove the high level of inorganic nutrients contained in the post–treatment domestic wastewater, which had been banned by the local government for release into the sea.

**Figure 1:**
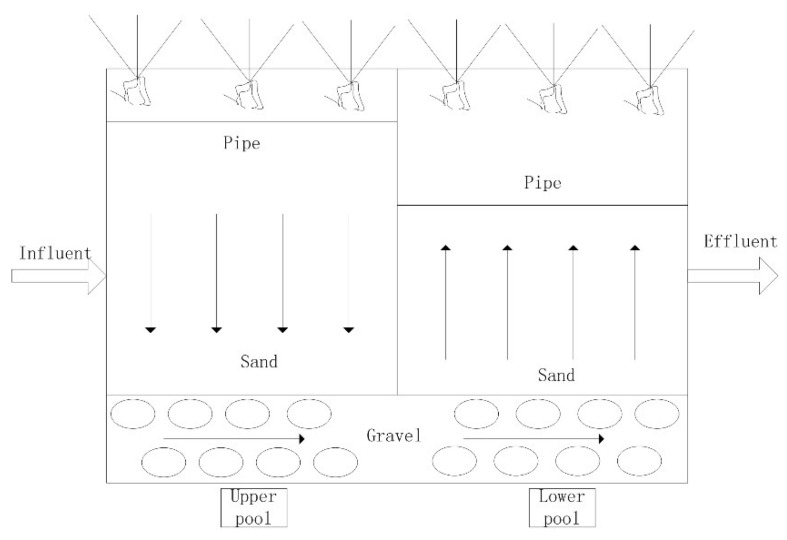
Schematic diagram of the constructed wetland (CW) in Zhoushan, China.

The structure of the CW is of an integrated vertical flow constructed wetland (Figure 1). The CW system was established with two layers of filters: 0.5 m layer of gravel with a grain diameter of 40–70 mm followed by a 0.4 m layer of coarse sand with a 1–5 mm grain diameter at the top. This system was fed with domestic wastewater after pre-treatment. The sewage evenly flows into the upper pool of the CW through the distribution pipe. The distribution pipe is evenly arranged under the surface of the upper pool sand substrate, and the catchment pipe of the lower pool is buried in the sand substrate (Figure 1). This kind of CW structure has no water on the surface, and most of the area is close to the meso-environment. It is suitable for the growth of more plant species, so it has a better treatment effect and landscape function.

### 2.2. Cultivation of Plants in CW for Biofuel Production

We conducted trials on the CW in Zhoushan in 2016–2017 to obtain field-scale biomass production information. We established 12 plots, each 2.0 × 2.5 m, for bioenergy production experiments. We selected 7 species with relatively high biomass productivity [12], including main macrophytes used in the CW, i.e., *Phragmites australis*, *Typha latifolia*, *Cyperus papyrus*, *Canna indica*, *Phalaris arundinacea*, *Arundo donax*, and *Glyceria maxima*. In April every year, all plant species were transplanted at a density of 10 seedlings m^−2^, with initial seedling heights of 20–30 cm. All plots were hand-weeded during the summer in 2016 and 2017. For measuring the aboveground biomass production, a 0.5 × 2.0 m strip was placed in the centre of each plot to avoid edge effects, following the method used in LIHD grassland experiments [5]. We then cut the plants at 5 cm above the surface of the substrate at the end of September in 2016 and 2017. We sorted the harvested plant material by species, dried the samples at 65 °C to a constant weight, and then scaled the weight to the full 2.0 × 2.5 m plots.

Before the plant harvested, the gas samples were collected by the static chamber method [19] for N_2_O and CH_4_ gas measurement. The chamber (diameter = 31 cm, height = 32 cm) was opaque and made of high-density polyvinyl chloride materials. Gas sampling was conducted simultaneously for each plant in the 0.5 × 2.0 m strip in April of 2016 and 2017. Gas collection was carried out at intervals of 30 min from 08:00 to 10:00 to ensure the temperature was relatively consistent (the temperature was constant at 28.1 °C). A gas sample from each microcosm was collected into a 100 mL vacuum sampling bag (Plasticgas, Delin Company, Nanjing, China) using a polyurethane syringe. The concentrations of CH_4_ and N_2_O in the gas sampling bags were determined using a gas chromatograph (Agilent-7820, Palo Alto, CA, USA) with a detector (a flame ionization detector and an electron capture detector, respectively) and a 3 m Poropak Q column.

### 2.3. Life Cycle Assessment (LCA) 

Based on ISO 14040 and 14044, LCA is a tool for evaluating environmental aspects and investigating the performance of different productions, such as agriculture production, and comprises of the following four steps: Goal definition and scoping, life cycle inventory, impact assessment, and life cycle results interpretation. In our study, LCA was performed to estimate the energy balance and GHG emissions during biomass production in the CW, from seedling growth to harvest, and conversion to cellulosic ethanol, based on all stages of construction and operation.

#### 2.3.1. Life Cycle Assessment of Energy Balance

Life cycle analysis was used to evaluate the net energy balance for the CW based on a previous method used for switchgrass and LIHD grassland [4,5]. The energy inputs for the CW bioenergy system included the energy used during the construction of CW infrastructure, material production and transportation, labor in construction work, planting of seedlings, operations for wastewater treatment, energy consumption during biomass growth, harvesting, transportation to factories, and conversion of biomass to biofuel for biorefinery. The energy output of the CW biofuel production was calculated as biomass converted into ethanol; biomass converted to ethanol via cellulose digestion to sugars followed by fermentation and distillation. The energy output of biofuel production includes the combustible energy of biofuels themselves. We transformed the aboveground biomass production per hectare into energy output per hectare for the CW plant based on: 0.38 L ethanol kg^−1^ harvested biomass and 21.5 MJ (1 MJ = 10^6^ J) L^−1^ ethanol [4].

To compare the energy balance in a wastewater treatment process, we contrasted the CW and WTP. We selected a WTP in the same region as comparison, located in Zhoushan, Zhejiang Province. The daily treatment design scale of the Zhoushan WTP is 150,000 cubic meters per day. Through visitations and investigative research, we collected the related parameter data of the WTP’s construction and operation process. For the WTP, the energy consumed in wastewater treatment includes initial energy consumption in construction work and successive energy consumption during operation, while there were no energy outputs. We assume that the CWs and WTPs have a life span of 20 years, thus annual energy consumption in construction work was calculated as divided by 20 years.

#### 2.3.2. Life Cycle Assessment of GHG emission

**Figure 2:**
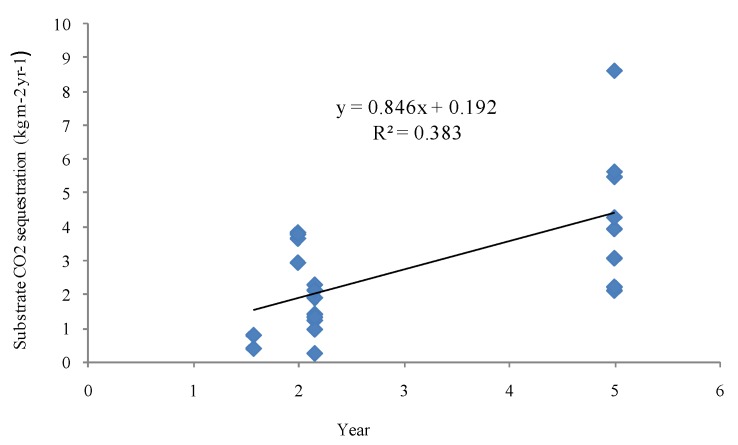
Soil organic carbon dioxide to ~0.3 m soil depth for the CW plant.

During wastewater treatment and biofuel production, three major species of GHG (CO_2_, CH_4_, and N_2_O) emissions were recorded as the main environmental effect. When measuring the life-cycle environmental CO_2_ impacts of CW biofuel production, we included the total CO_2_ emissions from biofuel production as well as the CW ecosystem CO_2_ sequestration. We took CO_2_ storage in substrate as the CW ecosystem CO_2_ sequestration however, aboveground biomass carbon incorporated into plants is harvested at least once per year and is released back as CO_2_ into the atmosphere through the food web within the year. This implies that this increasing crop biomass does not contribute to a net long-term sink [20]. Considering that substrate C data needs to be measured for many years, in order to avoid the error caused by only measuring substrate C data of constructed wetlands for two years in this study, we adopted the calculation method of CO_2_ storage in substrate. Annual carbon storage in substrate of the CW was a linear function of operation years [21] (Figure 2). The release of CO_2_ from CW biofuel production includes seedling plantation, labor for harvest, feedstock transportation, conversion of crop to biofuel in a refinery factory, electricity, and labor for operating the CW. Thus, net ecosystem CO_2_ sequestration means the CW ecosystem CO_2_ sequestration minus CO_2_ released from CW biofuel production. 

Together, with CH_4_ and N_2_O emission (seen in Section 2.2), we collected net GHG from biofuel production, which is summed up. Thus, biofuels can be carbon neutral (no net effect on atmospheric CO_2_ and other GHG), carbon negative (net reduction in GHG), or carbon source (net increase in GHG) [5], depending on how much CO_2_ and other greenhouse gases, expressed as CO_2_ equivalents (CH_4_ has a global warming potential of 25 relative to CO_2_ and N_2_O has a global warming potential of 298 relative to CO_2_, IPCC 5th report) [22], are removed from or released into the atmosphere during plant growth as well as how much fossil CO_2_ is released in CW biofuel production.

For a comparison of CO_2_ released from the CW and WTP, we included the construction and operation stage for management as well. We transformed the estimates of per hectare energy use into per hectare CO_2_ emission for an emission factor of 81.56 g CO_2_ MJ^−1^ [6].

### 2.4. Statistical Analysis

All variance analysis and linear regression analysis were performed in SPSS 13.0 (SPSS Inc., Chicago, IL, USA), with a statistical significance of *a* = 0.05. All data are expressed as mean + standard error (SE).

## 3. Results and Discussion

### 3.1. Biomass Production by the CW

Plant aboveground biomass in the Zhoushan CW ranged from 565 kg ha^−1^ year^−1^ to 90,000 DW kg ha^−1^ year^−1^ (Table 1). The estimated peak aboveground biomass yield of the CWs in our experiment field in the southeast region of China is as high as ~90,000 DW kg ha^−1^ year^−1^, which reached a higher line to previous biofuel feedstocks’ potential productivity (Table 1) and is similar to Napier grass (*Pennisetum purpureum*), which is 88,000 kg ha^−1^ year^−1^ in the USA, and slightly lower than 100,000 kg ha^−1^ year^−1^ for natural stands of *Echinochloa polystachya* on the Amazon floodplain, which is almost the highest level of biomass production of biofuel feedstock [23]. 

The average biomass value for the CW plant collected in our study is 37,813 DW kg ha^−1^ year^−1^. For first-generation biofuel production, aboveground biomass production for corn in the USA is 3000–7000 kg ha^−1^ year^−1^ [26], with the highest annual dry matter production level being 9296 kg ha^−1^ year^−1^ [3]. Average aboveground biomass production for soybean is 2661 kg ha^−1^ year^−1^ [3]. Switchgrass is the most popular second-generation biofuel feedstock, with an aboveground biomass of 5200–11,000 kg ha^−1^ year^−1^ [4]. Biomass derived from LIHD mixtures of native grassland perennials and wood waste can provide 3682–6000 kg ha^−1^ year^−1^ and 3900–7800 kg ha^−1^ year^−1^ [5,30]. Thus, biomass production in the Zhoushan CW is the highest among the traditional biofuel feedstock we collected in the literature.

### 3.2. Energy Balances of the CW in Producing Biofuel

**Figure 3:**
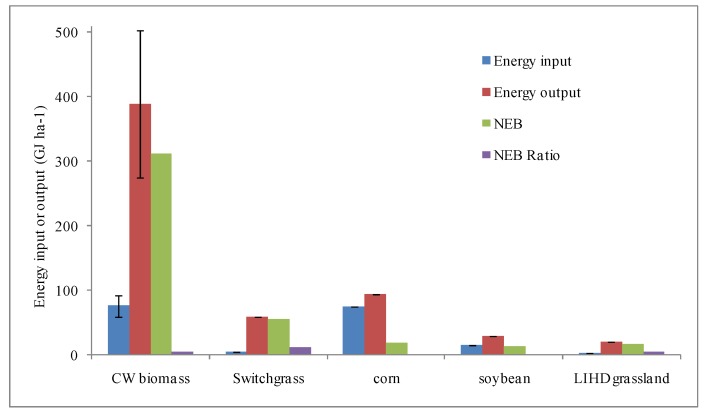
Comparison of energy input, output, NEB (Net Energy Balance), and the NEB ratio for five major biofuels: the CW, switchgrass, corn, soybean, and low-input high-diversity (LIHD) grassland. Note: Energy output for switchgrass, corn, soybean, and LIHD grassland is 60 GJ ha^−1^ [4], 94.5 GJ ha^−1^ [5], 29.9 GJ ha^−1^ [5], and 21.8 GJ ha^−1^ [5], respectively. NEB = energy output − energy input. The NEB ratio = energy output/energy input.

Figure 3 reports the comparison of energy input and output evaluated for these five biofuels: CW biomass, switchgrass, corn, soybean, and LIHD grassland. For the CW in Zhoushan, the average energy input was 76.9 GJ ha^−1^ year^−1^, the energy output was 389.2 GJ ha^−1^ year^−1^, NEB (Net Energy Balance) was 312.4 GJ ha^−1^ year^−1^, and the NEB ratio (energy output/energy input) is 5.06 (Figure 3). Both the energy input and output of the CW were highest among biofuel production systems such as switchgrass, corn, soybean, and LIHD grassland. However, the NEB ratio of the CW ranks in the middle level (5.06) with switchgrass having a ratio of 12.8, the LIHD grassland a ratio of 5.44, soybean a ratio of 1.93, and corn a ratio of 1.25.

The first-generation biofuel production is nitrogen intensified. The applied N fertilizer was as high as 146 kg/ha for corn gain ethanol production [3]. Second-generation biofuel used less N-fertilizer. For example, switchgrass needed 74 kg/ha [4], while the LIHD grassland needed only 2.2 kg/ha because legumes in high-diversity species mixtures may eliminate the need for nitrogen fertilization [5]. However, if the CW is taken as a new generation of biofuel production, it will become N-negative (no extra N supply), since when N-rich wastewater passes through the CW bed, N was retended by the plants and used as biofuel production.

### 3.3. Life-Cycle Environmental Effects of the CW

CO_2_ sequestration in soil for the CW is an average of 31 Mg (1 Mg =10^6^ g) ha^−1^ year^−1^, which exceeds the fossil CO_2_ released during CW biofuel production (1.2 Mg CO_2_ ha^−1^ year^−1^) (Table 2). For the LIHD grassland, fossil fuel combustion during agriculture, transportation, and processing was 0.3 Mg ha^−1^ year^−1^ of CO_2_, with net life cycle sequestration of 4.4 Mg ha^−1^ year^−1^ of CO_2_ for the first decade and an estimated 2.7 to 3 Mg ha^−1^ year^−1^ for subsequent decades [5]. For switchgrass grown in the northern plains of the USA, net CO_2_ sequestration exceeded 11.7 Mg CO_2_ ha^−1^ year^−1^ than the LIHD grassland, but was 14 Mg CO_2_ ha^−1^ year^−1^ lower than our CW biofuel production system (Table 2).

N incorporation from a wastewater influent for the CW and N fertilization of plant biomass into soil for corn, soybean, and switchgrass production systems, can cause microbially mediated production and the release of N_2_O, which is a potent GHG [11]. In addition, the CW had been regarded as a large contributor to sources of methane (CH_4_) [22]. Our results show that CH_4_ emission was 17.2 Mg CO_2_ eq. ha^−1^ year^−1^, whereas the LIHD grassland could mitigate CH_4_ (Table 2). N_2_O emission in the CW is 23 times more than the LIHD grassland.

In summary, GWP (global warming potential) is used for measuring the greenhouse effect. On a 100-year time horizon, CH_4_ has a global warming potential of 23 relative to CO_2_, and N_2_O has a global warming potential of 296 relative to CO_2_ [22]. Net GHG reduction from the CW biofuel production system is 8.8 Mg CO_2_ equivalent ha^−1^ year^−1^, which is twice more than the LIHD grassland (3.7 Mg CO_2_ eq. ha^−1^ year^−1^) (Table 2).

### 3.4. Replacing the Wastewater Treatment Approach by the CW

The CW was originally designed for wastewater treatment, while the current mainstream treatment approach was WTP. For the purposes of examining this issue in a contrasting context with the ‘have to’ cost of wastewater treatment, we made the comparison between the CW and WTP. 

For the comparison, the energy input, construction material, construction work, and operation facilities are included. Our analyses show that the CW results in a positive NEB while the WTP has a negative NEB since there was no energy output, with an energy input of 7442.5 GJ ha^−1^ year^−1^ (Table 3). The energy input for the WTP is 40 times more than the CW (Table 3). In this way, used as a wastewater treatment methods similar to the WTP, the CW could save energy 7649.1 GJ ha^−1^ year^−1^ when compared with the WTP on average. The saved energy could be viewed as benefit generated from the CW.

Across full life cycle analysis, C in the wastewater influent and effluent of the CW and WTP are biogenic, and was not included in the life cycle analysis (IPCC). Thus, CO_2_ emission during the construction and operation stages for the CW and WTP is the objective considered for comparison. Results show that on average the CW emitted 10.7 Mg CO_2_ ha^−1^ year^−1^, whereas the WTP emitted 607.3 Mg CO_2_ ha^−1^ year^−1^ (Table 4). In a scenario analysis: If the current mainstream wastewater treatment WTP is replaced by the CW, i.e., setting CO_2_ emission during the wastewater treatment in WTP as baseline, the CW will emit 596.6 Mg CO_2_ ha^−1^ year^−1^ less than the WTP. For a rough estimate, if we suppose there is 4.6 × 10^5^ ha of CW in China, it could reduce 4.9 Tg CO_2_ year^−1^. The use of fossil fuels in China produced CO_2_ emissions of 5.5 Pg into the atmosphere in 2016 [20]. Thus, using CW rather than WTP for wastewater treatment in China could reduce 0.1% of fossil fuel CO_2_ emission.

CH_4_ emission of CW is three orders of magnitude lower than the WTP (Table 3). N_2_O emission of the CW is 0.04 Mg ha^−1^ year^−1^, which is two orders of magnitude lower than that from the WTP (Table 3). In summary, if the WTP is replaced by the CW, the CW will emit 86,045.4 Mg CO_2_ eq. ha^−1^ year^−1^ less than the WTP (Table 3). From a biomass energy production perspective, the CW sequesters 5.1 Mg CO_2_ eq. ha^−1^ year^−1^ more than in a LIHD grassland (Table 2). Thus, if the CW replaces the WTP in the future as the main wastewater treatment approach, the total GWP reduction intensity for the CW will be 86,050.5 Mg CO_2_ eq. ha^−1^ year^−1^.

### 3.5. Economic and Social Feasibility of Developing a CW

The cost of building a CW is only one-third to one-half of the cost of a WTP [12]. Moreover, the costs of operation and maintenance are less than one-tenths than that of WTPs [12]. From an economic point of view, constructing a CW is more feasible, especially for developing countries.

**Figure 4:**
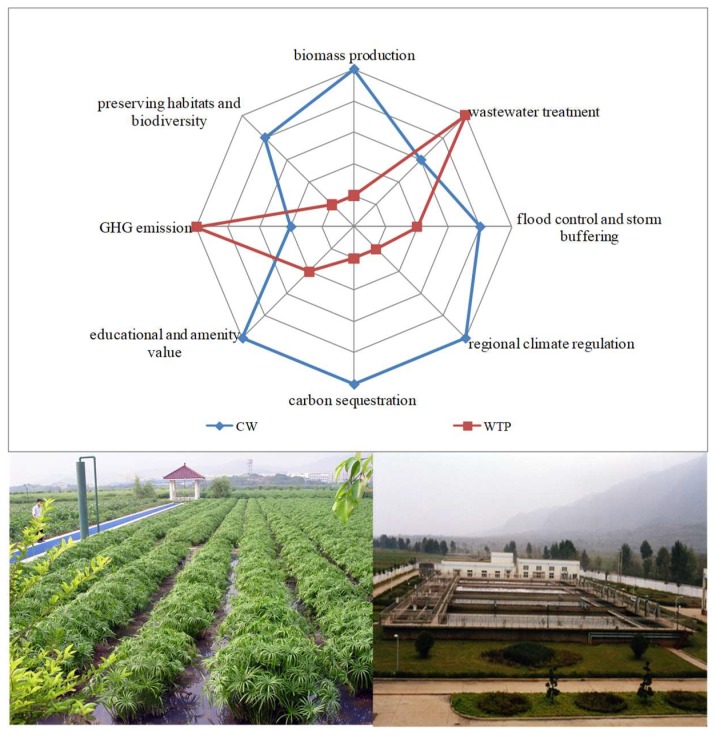
Conceptual frameworks comparing ecosystem services of a CW (blue line, left picture) and a WTP (wastewater treatment plant) (red line, right picture). The condition of each service in the diagrams is indicated along each ‘petal’ to illustrate the provisioning of multiple ecosystem services under different land use regimes. The value for radiation point of each ecosystem service is increased from the inside to the outside.

Although a CW requires lower investment and operation costs than a WTP, the land area needed is larger [11,13]. CWs require an area nine times larger than a WTP in order to remove an equal quantity of N considering total N effluent reach the limit 15–20 mg/L restricted by GB17918-2002 [31]. The high land requirement for CWs is the main barrier for expanding the application of a CW and it cannot be applied in densely populated areas where land prices are often too high. Additionally, CW can be constructed in many places because there is no need to use WTP to collect wastewater for treatment. The feasible land includes marginal land, barren soil sand, ribbing land etc. For example, a CW can be constructed around rural cropland to intercept N runoff from agricultural sources. Moreover, a CW can provide additional ecosystem service benefits, such as biomass production, carbon sequestration, reusable water supply, regional climate regulation, habitat conservation, and educational and recreational usage (Figure 4). Traditional WTP systems deliver better treatment functions, but provide almost no other services, except as a reusable water supply, flood control, and educational usage. In such a way the disadvantage of requiring a large amount of land for a CW can be mitigated.

## 4. Uncertainty Analysis

It remains difficult to acquire accurate estimates due to large variations in climate, soil characteristics, and operation practices in terms of evaluating the net energy balance and carbon sequestration potential of the CW biofuel production system on both regional and national scales. Uncertainty assessments, therefore, are important for policy makers since they should be aware of the risks in promoting this kind of wastewater treatment approach. 

In this study, we used actual above-ground biomass to determine biofuel energy outputs. The higher biomass we are reporting in comparison to the estimates reported previously clearly highlight discrepancies that can occur when plants are grown in a CW in a sub-tropic zone in China. In CWs throughout the world, biomass production should follow a low-high latitude gradient, with the highest levels of biomass occurring in low latitudes. However, no such correlation occurred in our analysis, and the highest biomass occurred in the near 30° latitude. This is because biomass productions reported worldwide are almost all in developed countries such as Europe and America, with little reports existing in tropical zone [12]. Moreover, plants used frequently in a CW are low-biomass yielding plants that decrease the cost of labor and waste disposal, including *Phragmites australis*, *Typha latifolia*, and *Canna indica*. The plant selection of a CW in the future can be diversified and improved since biomass can be used for biofuel production. Aboveground biomass production in a sub-tropic region we used in this study for evaluating biofuel output throughout China, which may be overestimated because it could not reach such a high value in the cold of North China. In addition, some factors of uncertainties include the climate, substrate texture, and wastewater conditions, which can also lead to variations between different CW experiments.

In this study, the change of soil organic carbon dioxide was only inferred from the simple linear regressions and this simplification may result in some potential uncertainties. However, net ecosystem carbon dioxide sequestration was 4.4 Mg ha^−1^ year^−1^ of carbon dioxide in the soil and roots of the LIHD grassland (Table 2), and we only used soil organic carbon dioxide in the CW for comparison, thus it can be inferred that we may underestimate the net ecosystem carbon dioxide sequestration.

## 5. Conclusions

Using CWs to simultaneously treat wastewater and produce biofuels takes advantage of the excessive waste of N, does not need additional N fertilization, and saves considerable energy input, when compared to other biofuel production systems. Based on our analysis, CWs can produce more renewable energy than is consumed in their production, and provides significant environmental benefits. They have the potential to be another source of feedstock for biofuels. Since CW biomass can be produced on marginal lands and green space land, CW biofuels need neither compete for fertile soils with food production nor encourage ecosystem destruction. If we take a conventional WTP as the baseline, we find that the CW exhibits low GHG emission potential. The CH_4_, N_2_O, and fossil fuel CO_2_ emissions for CWs were much lower than those of a WTP. Concerns for its potential to be widely adopted by people is that CWs are scattered in space, especially in rural areas in China, which make it difficult to collect CW plant biomass. Our proposed strategy of using CWs for biofuel production could be enhanced by the development of a centralized rural sewage treatment system in rural areas in the future.

## Figures and Tables

**Table 1:** Aboveground biomass production of current or potential biofuel plants.

Plant	Latin Name	Aboveground Biomass (kg ha^−1^ year^−1^)	Peak Aboveground Biomass (kg ha^−1^ year^−1^)	Reference
CW ^1^ plant	*Phragmites australis, Typha latifolia, Arundo donax,* et al.	37,813	90,000	Our study
Switchgrass	*Panicum virgatum* L.	5200–11,100	11,100	[4,24]
Miscanthus	*Miscanthus x giganteus*	15,000–40,000	40,000	[24,25]
Napier grass	*pennisetum purpureum*		88,000	[25]
	*Echinochloa polystachya*		100,000	
Poplar		5000–11,000	11,000	[25]
Agave spp.	*Agave tequiliana*	10,000–34,000	34,000	[25]
Sugarcane		10,000–11,000	11,000	[25]
Corn grain		3000–7000	9296	[3,26]
Soybean biodiesel		2661		[3]
Willow		16,000–18,000	18,000	[27,28]
LIHD ^2^ grassland		3682–6000		[5,25]
Wood waste		3900–7800		[29]
Municipal solid waste		2500–4600		[30]

Note: ^1^ CW refers to constructed wetland; ^2^ LIHD grassland refers to low-input high-diversity grassland.

**Table 2:** Net CO_2_ sequestration, net greenhouse gas (GHG) reduction for the CW, the LIHD grassland, and switchgrass (positive value means sequestration, while negative value indicates release, unit: Mg CO_2_ eq. ha^−1^ year^−1^).

Item	CO_2_ Soil/Root Sequestration	N_2_O Emission	CH_4_ Emission	CO_2_ Release from Biomass Production ^1^	Net CO_2_ Sequestration ^2^	Net GHG Reduction from Biofuel Production ^3^
CW biofuel	31.0	−3.7	−17.2	−1.2	29.8	8.8
LIHD grassland	4.0 ^4^	−0.2	0.2	−0.3	4.1	3.7
Switchgrass	16.2 ^5^	-	-	−0.4	15.8	-

Note: ^1^ CO_2_ released during biomass production, which includes seedling plantation, harvesting, and feedstock transportation; ^2^ Net CO_2_ sequestration = CO_2_ storage in substrate − CO_2_ released during biomass production; ^3^ Net GHG reduction from biofuel production = CO_2_ soil/root sequestration + N_2_O emission + CH_4_ emission + CO_2_ released from biomass production; ^4^ CO_2_ storage in soil for the LIHD grassland also includes root, data for the LIHD grassland in this line is from [5]; ^5^ C storage in soil profile is 4.42 Mg ha^−1^ year^−1^ [4], and was converted to 16.2 Mg CO_2_ ha^−1^ year^−1^. Data for switchgrass in this line is from [4].

**Table 3:** Net energy balance, net GHG reduction for the CW and wastewater treatment plant (WTP) (positive values means sequestration, negative values indicates release).

Item	Environmental Effect (Mg CO_2_ Equivalent ha^−1^ year^−1^)				Energy Effect (GJ ha^−1^)		
	CO_2_^1^	CH_4_	N_2_O	GWP ^4^	Energy Input	Energy Output	NEB ^5^
CW	−10.7	−17.2	−3.7	−31.7	182.6	389.2	206.6
WTP	−607.3	−84,957.4 ^2^	−481.0 ^3^	−86,045.4	7442.5	0.0	−7442.5

Note: ^1^ CO_2_ includes the construction and operation stage, value is seen in Table 4; ^2^ data for CH_4_ emission of the WTP is collected from literature [5]; ^3^ data for N_2_O emission of the WTP is collected from literature [5]; ^4^ GWP refers to Global warming potential; ^5^ NEB refers to Net Energy Balance.

**Table 4:** Energy consumption (unit: GJ ha^−1^ year^−1^) and CO_2_ emission (unit: Mg CO_2_ ha^−1^ year ^−1^) of total input for the WTP and the CW for wastewater treatment.

Item	Energy Consumption^1^ (GJ ha^−1^ year^−1^)			CO_2_ Emission (Mg CO_2_ ha^−1^ year^−1^)		
	SSF-CW ^2^	SF-CW ^3^	WTP	SSF-CW	SF-CW	WTP
Construction	124.4	21.6	1733.9	10.1	1.8	141.5
construction material	85.1	7.1	886.8	6.9	0.6	72.4
steel	0.5	—	719.4	0.0	—	58.7
cement	33.0	6.6	77.6	2.7	0.5	6.3
metal pipe	—	—	62.4	—	—	5.1
timber	—	—	2.2	—	—	0.2
gravel	17.7	—	22.4	1.4	—	1.8
sand	4.2	—	2.9	0.3	—	0.2
PC ^4^ liner	20.7	—	—	1.7	—	—
PE ^5^ pipe	4.6	0.5	—	0.4	0.0	—
geotextile	4.5	—	—	0.4	—	—
Transportation	27.5	2.7	55.7	2.2	0.2	4.5
Construction work	—	—	791.4	—	—	64.6
Seedling plant	11.8	11.8	—	1.0	1.0	—
Operation	58.2	58.2	5708.6	4.8	4.8	465.8
Electricity and fuel	32.3	32.3	3193.1	2.6	2.6	260.6
Chemical	—	—	2449.6	—	—	199.9
Labor	25.9	25.9	65.9	2.1	2.1	5.4
Total input	182.6	79.9	7442.5	14.9	6.5	607.3

Note: ^1^ Energy consumption (GJ ha^−1^ year^−1^) is converted to CO_2_ emission (Mg CO_2_ ha^−1^ year^−1^) and is by the emission factor: 81.6 g CO_2_ eq. MJ^−1^ ethanol produced. ^2^ SSF refers to subsurface; ^3^ SF refers to surface; ^4^ PC refers to polycarbonate; ^5^ PE refers to polyethylene.

## References

[1] Gu B.J., Ju X.T., Wu Y.Y., Erisman J.W., Bleeker A., Reis S., Sutton M.A., Lam S.K., Smith P., Oenema O. (2018). Cleaning up nitrogen pollution may reduce future carbon sinks. Global Environ. Chang..

[2] Goldemberg J. (2007). Ethanol for a sustainable energy future. Science.

[3] Hill J., Nelson E., Tilman D., Polasky S., Tiffany D. (2006). Environmental, economic, and energetic costs and benefits of biodiesel and ethanol biofuels. Proc. Natl. Acad. Sci. USA.

[4] Schmer M.R., Vogel K.P., Mitchell R.B., Perrin R.K. (2008). Net energy of cellulosic ethanol from switchgrass. Proc. Natl. Acad. Sci. USA.

[5] Tilman D., Hill J., Lehman C. (2006). Carbon-negative biofuels from low-input high-diversity grassland biomass. Science.

[6] Zhou X., Xiao B., Ochieng R.M., Yang J. (2009). Utilization of carbon-negative biofuels from low-input high-diversity grassland biomass for energy in China. Renew. Sustain. Energy Rev..

[7] Tilman D., Socolow R., Foley J., Hill J., Larson E., Lynd L., Pacala S., Reilly J., Searchinger T., Somerville C., Williams R. (2009). Beneficial biofuels—The food, energy, and environment trilemma. Science.

[8] Vymazal J. (2007). Removal of nutrients in various types of constructed wetlands. Sci. Total Environ..

[9] Tanner C.C. (1996). Plants for constructed wetland treatment systems—A comparison of the growth and nutrient uptake of eight emergent species. Ecol. Eng..

[10] Ciria M.P., Solano M.L., Soriano P. (2005). Role of macrophyte Typha Latifolia in a constructed wetland for wastewater treatment and assessment of its potential as a biomass fuel. Biosyst. Eng..

[11] Kivaisi A.K. (2001). The potential for constructed wetlands for wastewater treatment and reuse in developing countries: A review. Ecol. Eng..

[12] Liu D., Ge Y., Chang J., Peng C.H., Gu B.H., Chan G.Y.S., Wu X.F. (2009). Constructed wetlands in China: Recent developments and future challenges. Front. Ecol. Environ..

[13] Zhang D.Q., Gersberg R.M., Keat T.S. (2009). Constructed wetlands in China. Ecol. Eng..

[14] Zhu S.X., Ge H.L., Ge Y., Cao Q.J., Liu D., Chang J., Zhang C.B., Gu B.J., Chang S.X. (2010). Effects of plant diversity on biomass production and substrate nitrogen in a subsurface vertical flow constructed wetland. Ecol. Eng..

[15] Zhang C.B., Wang J., Liu L.W., Zhu S.X., Liu D., Chang S.X., Chang J., Ge Y. (2010). Effects of plant diversity on nutrient retention and enzyme activities in a full-scale constructed wetland. Bioresour. Technol..

[16] Chang J., Liu D., Cao H.Q., Chang S.X., Wang X.Y., Huang C.C., Ge Y. (2009). NO_3_^−^/NH_4_^+^ ratios affect the growth and N removal ability of *Acorus calamus* and *Iris pseudacorus* in a hydroponic system. Aquat. Bot..

[17] Yang W., Chang J., Xu B., Peng C.H., Ge Y. (2008). Ecosystem service value assessment for constructed wetlands: A case study in Hangzhou, China. Ecol. Econ..

[18] Perbangkhem T., Polprasert C. (2010). Biomass production of papyrus (*Cyperus papyrus*) in constructed wetland treating low-strength domestic wastewater. Bioresour. Technol..

[19] Chang J., Fan X., Sun H.Y., Zhang C.B., Song C.C., Chang S.X., Gu B.J., Liu Y., Li D., Wang Y. (2014). Plant species richness enhances nitrous oxide emissions in microcosms of constructed wetlands. Ecol. Eng..

[20] Piao S.L., Fang J.Y., Ciais P., Peylin P., Huang Y., Sitch S., Wang T. (2009). The carbon balance of terrestrial ecosystems in China. Nature.

[21] Zhang C.B., Wang J., Liu W.L., Zhu S.X.L., Ge H.L., Chang S.X., Chang J., Ge Y. (2010). Effects of plant diversity on microbial biomass and community metabolic profiles in a full-scale constructed wetland. Ecol. Eng..

[22] Pachauri R.K., Meyer L.A., IPCC (Intergovernmental Panel on Climate Change), Core Writing Team (2014). Contribution of Working Groups I, II and III to the Fifth Assessment Report of the Intergovernmental Panel on Climate Change. Climate Change 2014: Synthesis Report.

[23] Bansal A.B., Illukpitiya P., Tegegne F., Singh S.P. (2016). Energy efficiency of ethanol production from cellulosic feedstock. Renew. Sustain Energy Rev..

[24] Smeets E.M.W., Lewandowski I.M., Faaij A.P.C. (2009). The economical and environmental performance of miscanthus and switchgrass production and supply chains in a European setting. Renew. Sustain Energy Rev..

[25] Somerville C., Youngs H., Taylor C., Davis S., Long S.P. (2010). Feedstocks for lignocellulosic biofuels. Science.

[26] González-García S., Mola-Yudego B., Murphy R.J. (2013). Life cycle assessment of potential energy uses for short rotation willow biomass in Sweden. Int. J. Life Cycle Ass..

[27] Börjesson P., Berndes G. (2006). The prospects for willow plantations for wastewater treatment in Sweden. Biomass Bioenergy.

[28] Shi Y., Ge Y., Chang J., Shao H.B., Tang Y.L. (2013). Garden waste biomass for renewable and sustainable energy production in China: Potential, challenges and development. Renew. Sustain Energy Rev..

[29] Brown S. (2010). Putting the landfill energy myth to rest. BioCycle.

[30] Robertson G., Paul E., Harwood R. (2000). Greenhouse gases in intensive agriculture: Contributions of individual gases to the radioactive forces of the atmosphere. Science.

[31] NSPRC (National Standards of the People’s Republic of China) (2002). Discharge Standard of Pollutants for Municipal Wastewater Treatment Plant. https://www.ecolex.org/details/legislation/discharge-standard-of-pollutants-for-municipal-wastewater-treatment-plant-national-standard-gb-18918-2002-lex-faoc136765/.

